# *Laubuka tenella*, a new species of cyprinid fish from southeastern Bangladesh and southwestern Myanmar (Teleostei, Cyprinidae, Danioninae)

**DOI:** 10.3897/zookeys.742.22510

**Published:** 2018-03-12

**Authors:** Sven O. Kullander, Md. Mizanur Rahman, Michael Norén, Abdur Rob Mollah

**Affiliations:** 1 Department of Zoology, Swedish Museum of Natural History, PO Box 50007, SE-104 05 Stockholm, Sweden; 2 Department of Zoology, University of Dhaka, Dhaka-1000, Bangladesh

**Keywords:** DNA barcode, morphometry, phylogeny, South Asia, taxonomy, vertebral counts

## Abstract

*Laubuka tenella* is a new species characterized by the colour pattern, consisting of short dark vertical bars anteriorly on the side, and a dark lateral band posteriorly on the side, combined with a relatively short pelvic fin and 29–30 lateral-line scales. It is separated from other *Laubuka* analysed by minimum 9 % uncorrected *p*-distance in the mitochondrial COI gene. The type series is composed of specimens from small streams in the Cox’s Bazar District in Bangladesh (the type locality), and the Thandwe River drainage in western Myanmar. *Laubuka brahmaputraensis* is strongly indicated to be a junior synonym of *L. laubuca*, the second known species of *Laubuka* in Bangladesh. *Eustira ceylonensis*, currently in the synonymy of *Devario malabaricus*, is a valid species of *Laubuka*.

## Introduction

Species of the tropical Asian cyprinid genus *Laubuka* Bleeker, 1860, are characterized by relatively small size, up to about 60 mm SL, a strongly compressed body and a keeled abdomen, a long and stiff falciform pectoral fin, and usually a long free distal portion of the first pelvic-fin ray (Pethiyagoda et al. 2008, Silas 1958). *Laubuka* resembles *Devario* Heckel, 1843, in presence of supraorbital and rostral neuromast-filled grooves, posteriorly situated dorsal fin, and presence of a dark blotch immediately posterior to the gill opening. Unlike *Devario*, most species of *Laubuka* show only indistinct markings, and barbels have not been recorded from *Laubuka*.

Species of *Laubuka* have been reported from southern Asia in Bangladesh, Cambodia, India, Indonesia, Laos, Malaysia, Myanmar, Pakistan, Sri Lanka, Vietnam, and Thailand (Silas 1958, Kottelat 2013). About 13 species are recognized at present (Pethiyagoda et al. 2008, Knight 2015, Lalramliana et al. 2017), but the genus has not been subject to revision since that of Silas (1958), who employed a very different concept of taxa currently in *Laubuka*. Banarescu (1968) examined and illustrated several species of *Laubuka*, but his paper is mainly concerned with the description of a new subgenus, *Malayochela*, monotypic with *M. maassii* (Weber & de Beaufort, 1912). Banarescu’s concept of the other species of *Laubuka* is similar to that of Silas (1958). Several of the species presently in *Laubuka* were previously, e.g., in Silas (1958) and Banarescu (1968), in the genus *Chela* (Hamilton, 1822), from which they were separated by Pethiyagoda et al. (2008). Species diversity in *Chela* was addressed by Knight and Rema Devi (2014), who recognized two valid species in that genus. Another genus previously included as a subgenus of *Chela* is *Neochela* Silas, 1958, containing only the miniature species *N. dadiburjori* (Menon, 1952). *Malayochela* was considered to be a distinct genus by Pethiyagoda et al. (2008).

The type species of *Laubuka* is *Cyprinus laubuca* Hamilton, 1822, which was described from “ponds of the northern parts of Bengal” (Hamilton 1822), i.e., within the present state of West Bengal in India, and Bangladesh. Hamilton (in Day, 1877) reporting on surveys 1807–1813, specifically mentioned the species from Goalpara in Rangpur (currently Goalpara District, Assam, India, Brahmaputra basin), Mahananda River in Purniah (Purnea District, Bihar, Ganga basin), and in Gorakhpur District (Uttar Pradesh, Ganga basin), suggesting that he did not observe the species in localities within present-day Bangladesh. Hamilton left India in 1815 (Day, 1877), however, and would have had opportunities to make unrecorded observations of fishes. Silas (1958), followed by Talwar and Jhingran (1991), reported *L. laubuca* as distributed in Pakistan, India, Bangladesh, Sri Lanka, Myanmar, Malay Peninsula, and Sumatra. Recent publications only list specimens from India (Knight 2015, Pethiyagoda et al. 2008) and Bangladesh (Rahman and Chowdhuri 2007), but there is no recent revision covering this species. Silas’s (1958) concept of *L. laubuca* included the southeast Asian *L. siamensis* Fowler, 1939, and the Sri Lankan *L. lankensis* (Deraniyagala, 1960), revalidated by Pethiyagoda et al. (2008), and would have accommodated also the three Sri Lankan species described by Pethiyagoda et al. (2008) as *L. insularis*, *L. ruhuna*, and *L. varuna*.

Despite previous records from Bangladesh, we were unable to find *Laubuka* in natural habitats in the Meghna, eastern Brahmaputra, and Karnafuli drainages in Bangladesh During field work 2014–2016. *Laubuka laubuca* was, however, present in small numbers in aquarium shops in Dhaka, and shop owners said that they had been caught locally. In 2015, we collected specimens of *Laubuka* from streams near Cox’s Bazar and Teknaf in extreme southeastern Bangladesh. These samples represent a species very different from *L. laubuca*, but morphologically indistinguishable from a sample of *Laubuka* from a stream on the western slope of the Rakhine Yoma in Myanmar. This paper is dedicated to the description and diagnosis of this new species.

## Materials and methods

Specimens used were available in museums, purchased from fishermen or markets; or collected in the wild using beach seine or hand net and euthanized through immersion in buffered tricaine-methanesulphonate (MS 222) until cessation of opercular movements plus an additional 30 minutes, in accordance with permits from the Swedish Environmental Protection Agency (dnr 412-7233-08 Nv) and the Stockholm Ethical Committee of the Swedish Board of Agriculture (dnr N 85/15). Collecting in Bangladesh was conducted under a permit to the University of Dhaka. Voucher specimens are deposited in the collections of The Natural History Museum, London (**BMNH**), University of Dhaka, Dhaka (**DU**), Museum of Zoology, Lund University (**MZLU**), and the Swedish Museum of Natural History, Stockholm (**NRM**).

Measurements and counts were taken as described by Fang (1997) with the exception that body depth was taken at level of the origin of the anal-fin, which is very slightly anterior to the origin of the dorsal fin. Counts of lateral-line scales do not include perforated scales on the caudal-fin base. The last two dorsal- and anal-fin rays share the same proximal pterygiophore and may appear as a single ray; these two rays are counted as 1½. Vertebral counts are given as precaudal+caudal, where the first vertebra bearing a haemal spine (anterior to the first long anal-fin pterygiophore) was recorded as the first caudal vertebra. Digital X-radiographs made with a Kevex 130kVP microfocus X-ray source and a Samsung/Rayence 17×17 inch DR panel. Statistics were calculated using SYSTAT v. 13 (Systat Software 2009).

For the genetic analysis, 655 basepairs from the 5’ end of the mitochondrial cytochrome-oxidase subunit 1 (COI, or COX1) gene were sequenced from seven morphologically identified specimens of *Laubuka* plus two specimens of the closely related *Chela cachius* (Hamilton, 1822). DNA was extracted using a Thermo Scientific KingFisher Duo (Thermo Fisher Scientific, Waltham, USA) fully automated liquid-handling instrument, with the Thermo Scientific KingFisher Cell and Tissue DNA Kit (Thermo Fisher Scientific, Waltham, USA) with the recommended protocol. PCR were performed with the puReTaq Ready-To-Go PCR kit (Amersham Biosciences AB, Uppsala, Sweden). The COI fragment was amplified using the fish barcoding primers Fish-F1 and Fish-R1 [26], with the PCR cycling: 94 °C 4 min; 35 * (94 °C 30 s; 52 °C 30 s; 72 °C 30 s); 72 °C 8 min). The PCR products were checked on agarose gel, and purified by adding 5 µL of a mix consisting of 20 % Exonuclease I (EXO) and 80% FastAP Thermosensitive Alkaline Phosphatase (Fermentas/Thermo Fischer Scientific, Gothenburg, Sweden) to each 25 µl PCR reaction, incubated at 37 °C for 30 minutes, then heated to 80 °C for 15 minutes. Sequencing of both strands of all fragments was carried out by Macrogen Europe (Amstelveen, The Netherlands). Sequences were assembled, aligned, and proofread in Geneious version 10 (Kearse et al. 2012). Geneious was used to calculate genetic distances (uncorrected pairwise *p*-distance, as recommended by Srivathsan and Meier (2011)), and the Geneious plug-in Species Delimitation (Masters et al. 2011) was used to calculate the probability of reciprocal monophyly under a model of random coalescence. A phylogenetic hypothesis was constructed in MrBayes version 3.2 (Huelsenbeck and Ronquist 2001; Ronquist and Huelsenbeck 2003) (2 million generations, GTR + Γ + I model), data partitioned by codon position; samples were taken every 1000 generations, and the first 25 % of samples were discarded as ‘burn-in’. Convergence was checked with Tracer version 1.6 (Rambaut et al. 2014).

The distribution map was constructed using layers from Natural Earth (http://www.naturalearthdata.com).

**Comparative material. *Chela cachius***: DU 6116, 1, 40.9 mm SL. Bangladesh: Chittagong Division: Feni River drainage, Kohua River; M.M. Rahman, 29 May 2015. — NRM 40494. 4, 46.8–49.3 mm SL; India: Assam: about 100 km SE of Dibrugarh, small river falling into the Dibru River 3 km north of Digholtarang; F. Fang & A. Roos, 22 Jan 1998. — NRM 66988 (T10085), 1, 35.7 mm SL. Bangladesh: Dhaka Division: Padma River near Sreenagar, M.M. Rahman et al., 2 Dec 2014.

**Devario cf. malabaricus.** BMNH 1852.2.19.130–132, 1853.12.24.6, 1864.4–11.33; 6, 50.1– 67.2 mm SL; Ceylon.

***Laubuka laubuca***: Bangladesh: NRM 67315 (T10453), 1, 35.8 mm SL; NRM 67316 (T10454), 1, 31.9 mm SL; NRM 67317 (T10455), 1, 42.7 mm SL. Bangladesh, ornamental fish shops in Dhaka, said to be local fish; M.M. Rahman et al., 16 May 2015. India, Assam: NRM 40308, 1, 48.7 mm SL; NRM 40486, 1, 44.0 mm SL; NRM 40517, 55.6–58.3, Assam, about 100 km SE of Dibrugarh, rivulet falling into the Dibru River 3km N of Digholtarang, F. Fang & A. Roos, 22 Jan 1998. – NRM 44997 (T10965), 38.9 mm SL; Brahmaputra River at Tezpur, Sonitpur; H. Bleher, 28 Feb 2009. — NRM 52691, 1, 43.8 mm SL; Golaghat: Kaziranga, Deosor subdrainage; O. Åhlander et al., 24 Oct 2005. — NRM 57234, 2, 49.9–53.1 mm SL; Dibrugarh, Mr Kamal Lahkar’s home fish farm “Brahmaputra Aquaria”. F. Fang & A. Roos, 19 Jan 1998.

***Laubuka* sp.**: NRM 12218, 59.0 mm SL; India: Kerala: Meenachil River and adjacent canals, NW of Kottayam; E. Åhlander et al., 5–6 Dec 1987.

***Laubuka parafasciata* Lalramliana, Valalhlimpuia & Singh, 2017**: All from Myanmar, Rakhine State. BMNH 2017.8.2.2, 1, 45.6 mm SL; Kananme Chaung near Leldee village and Chaung Ma Gyi Chaung, Leldee village, 18°35.814'N, 94°22.853'E and 18°35.112'N, 94°22.182'E; R. Britz, 29 Nov 2009. — BMNH 2017.8.2.3-4, 2, 64.7–85.3 mm SL; Chaung Ma Gyi Chaung, Leldee village, N 18°35.112'N, 94°22.182’E; R. Britz, 28 Nov 2009. — BMNH 2017.8.2.21, 1, 37.8 mm SL; Kananmee Chaung; Ye Hein Htet, 3 Dec 2004. — BMNH 2017.8.2.23, 1, 46.9 mm SL; Ann Chaung near Ann; R. Britz, 24–25 Nov 2009. (Ann = 19°47’0"N, 94° 2'0"E).

***Laubuka insularis***: All from Sri Lanka. MZLU 962/5429, 2, 20.3–33.3 mm SL; Eastern: Rambukkan Oya River, 25 miles NE of Bibile. P. Brinck et al., 8 Mar 1962. (125). — MZLU 962/5442. 6, 32.9–44.0 mm SL; Eastern: Gal Oya River, 14 miles E of Bibile; P. Brinck et al., 8 Mar 1962. (123). — MZLU 962/5459, 1, 47.8 mm SL; North Central: Mahaweli River drainage: small stream 3 mi. S of Minneriya; P. Brinck et al., 11 Feb 1962 (67).

**Laubuka cf. varuna**: MZLU 962/5438, 4, 29.3–46.0 mm SL; Sri Lanka: Central: Talagala Oya stream at Pidurutalagala, 2 miles N of Nuwara-Eliya; P. Brinck et al., 4 Mar 1962. (116:I).

***Laubuka siamensis***: NRM 31223, 6. Viet Nam; Dong Nai River drainage, about 30 km ENE of Ho Chi MinhCity, Xa Trang Bom, road crossing small stream east of village, 10°57'1"N, 106°58'28"E; B. Björkegren, 25 Mar 1935. Information on *L. ruhuna*, *L. varuna*, *L. lankensis* from Pethiyagoda et al. (2008); on *L. khujairokensis* (Arunkumar, 2000) from Arunkumar (2000); on, *L. caeruleostigmata* (Smith, 1931) from Smith (1931); and on *L. trevori* Knight, 2015 and *L. latens* (Knight, 2015 from Knight (2015).

**GenBank Accession numbers.** New COI sequences generated for this paper are:

*Chela cachius*, DU 6116: MG895632; NRM 66988: MG895633.

*Laubuka laubuca*, NRM 67315: MG895634; NRM 67317: MG895635; NRM 44997: MG895636.

*Laubuka tenella*: NRM 67380: MG895640; DU 9008/NRM 67381: MG895637; NRM 67862: MG895639; DU 9006/NRM 67845: MG895638.

## Results

### Laubuka tenella sp. n.

Taxon classificationAnimaliaCypriniformesCyprinidae

http://zoobank.org/93EB4FA4-7823-4951-A3A7-A0F99BAF3672

Figs 1 , 2 , 3

#### Holotype.

**Figure 1.**
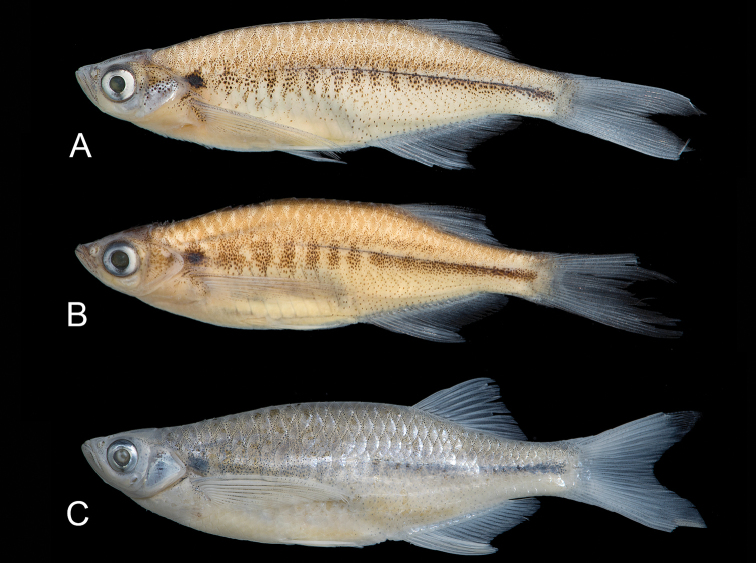
*Laubuka tenella*. **A** holotype, DU 9004, 42.1 mm SL. Bangladesh: Chittagong Division: Domdomia stream, 10 km north of Teknaf **B** *Laubuka tenella*, paratype, NRM 40813, 39.8 mm SL. Myanmar: Rakhine State: Thandwe River drainage, Nan Chaung, near Thandwe **C** *Laubuka tenella*, paratype, NRM 67380, 46.8 mm SL, preserved in 95% ethanol. Bangladesh: Chittagong Division, Majerchora stream, 10 km south of Cox’s Bazar.

(Fig. 1A). DU 9004, 42.1 mm SL. Bangladesh: Chittagong Division: Cox’s Bazar District: Naf River drainage, Domdomia stream, 10 km north of Teknaf town, 70 km south of Cox’s Bazar; 20°55'24"N, 92°15'47"E; M.M. Rahman et al., 9 May 2015.

#### Paratypes.

DU 9005, 6, 30.8–42.2 mm SL; DU 9006/NRM 67845, 1, 47.4 mm SL; DU 9007/NRM 67861, 1, 35.8 mm SL; NRM 67862, 1, 38.4 mm SL; NRM 69227, 1, 47.4 mm SL; NRM 68062, 7, 33.9–45.7 mm SL; same data as holotype. NRM 67380, 1, 46.8 mm SL; DU 9008/NRM 67381 , 1, not measured; Bangladesh, Chittagong Division: Bakkhali River drainage, Majerchora stream, 10 km south of Cox’s Bazar; 21°23'45"N, 92°0'16"E; M.M. Rahman et al., 8 May 2015. BMNH 2018.1.31.3–5, 3, 37.9 mm SL; NRM 40813, 15, 33.9–42.5 mm SL; NRM 69798, 10, 29.6–37.0 mm SL; Myanmar: Rakhine State; Thandwe River drainage: Nan Chaung, a stream at 3 km on road from Thandwe (market) to Ngapali; 18°27'8"N, 94°20'55"E; S.O. Kullander & R. Britz, 20 Mar 1998.

#### Diagnosis.

Distinguished from all other species of *Laubuka* except *L. insularis*, *L. lankensis*, *L. ruhuna*, and *L. varuna* by the colour pattern, including a dark stripe along the middle of the posterior third of the side or slightly shorter, anteriorly replaced by 6–11 short vertical bars (vs. presence of a dark stripe along the side but absence of bars in *L. fasciata*, *L. parafasciata*, and *L. trevori*; plain sides or presence of a very narrow posterior stripe in *L. khujairokensis*, *L. latens*, and *L. laubuca*; indistinct vertical bars anteriorly on the side, followed by a dark stripe ending in a triangular spot on the caudal-fin base in *L. siamensis*; a few dark bars present anteriorly on the side but lateral band absent in *L. caeruleostigmata*). Distinguished from *L. fasciata*, *L. latens* and *L. trevori* also by more dorsal-fin rays (ii.8½ vs. ii.7½) and from *L. siamensis* by the absence of a dark spot on the caudal-fin base. Distinguished from *L. insularis* by fewer scales in the lateral line (29–32 vs. 34–36), shorter pelvic fin (not reaching to vent, vs. reaching to bases of anal-fin rays 3–8); from *L. lankensis* by fewer scales in the lateral line (29–32 vs. 34–37); from *L. ruhuna* and *L. varuna* by the presence of an entire, narrow lateral band on the posterior third of the body, vs. a series of blotches along the side which may form a broad band extending anteriorly to about the middle of the side.

#### Description.

Elongate, strongly compressed laterally. Predorsal contour slightly ascending, levelling out close to dorsal fin-base, minor indentation at commencement of squamation. Dorsal-fin origin marking 2/3 of standard length, immediately posterior to vertical from anal-fin origin. Dorsal-fin base contour slanting, continuous with dorsal contour of caudal peduncle; caudal peduncle only slightly tapering caudad.

*Snout* shorter than orbital diameter, triangular in lateral aspect, rounded in dorsal aspect. Mouth terminal, lower jaw at about 50° angle, tip anterior to upper jaw, not quite reaching level of upper margin of orbit. Eyes large, lateral, in middle of head length, well visible in ventral aspect of head, in dorsal aspect only slightly. Anterior nostril tubular, opening anterolaterad. Supraorbital ending anteriorly in sharp point. Long shallow frontal and rostral neuromast grooves present. Barbels absent. Tubercles absent. Lower jaw with wide band of minute papillae, tentatively identified as neuromasts. Ventral outline more arched than dorsal; slanting about straight to under pectoral-fin base, posteriorly about straight horizontal to anal-fin insertion; anal-fin base contour straight ascending. Chest flat close to isthmus; from pectoral-fin base caudad to pelvic-fin base strongly compressed, posteriorly strongly compressed and with sharp keel formed by margins of opposed left and right side abdominal scales.

All *scales* cycloid, thin, transparent. Lateral line anteriorly descending for about five scales, posteriorly paralleling ventral outline, ending on lower half of caudal peduncle, continued by 1–2 scales basally on caudal fin. Single row of scales along base of anal fin. Elongate axillary pelvic-fin scale present. Lateral line scales 29 (1), 30 (2), 31 (7), 32 (3) in Cox’s Bazar specimens; 29 (1), 30 (5), 31 (7) in Rakhine specimens. Predorsal scales 16 (1), 17 (6), 18 (4) in Cox’s Bazar specimens; 16 (2), 17 (7), 18 (1) in Rakhine specimens. Circumpeduncular scales 12 (27). Scales between dorsal fin origin and lateral line ½6 (28); between lateral line and anal-fin origin 3 (28), of which distal scale part of abdominal keel.

*Dorsal-fin* origin at about posterior third of body, slightly posterior to origin of anal fin; with straight distal margin, rays gradually shorter from second unbranched ray to last ray. Dorsal-fin rays ii.8½ (29). Anal fin with longer base than dorsal fin; short rounded anterior lobe, posterior rays gradually shorter. Anal-fin rays iii.16½ (3), iii.17½ (3), iii.18½ (8) in Cox’ Bazar specimens; iii.18½ (3), iii.19½ (10, iii.20½ (2) in Rakhine specimens. Pectoral-fin long, falcate, unbranched ray longest or unbranched and first branched ray equally long, not reaching to vent; two large scales covering bases of branched fin rays and adjacent chest. Caudal fin forked to about middle of fin. Pectoral-fin rays i.10 (1), i.11 (10), i.11 (3) in Cox’s Bazar specimens; i.10 (4), i.11 (9), i.12 (2) in Rakhine specimens. Pelvic fin inserted slightly anterior to middle of side; short, unbranched ray longest with short prolongation, not reaching to vent. Pelvic-fin rays i.5 (1), i.6 (13) in Cox’s Bazar specimens; i.6 (15) in Rakhine specimens.

*Vertebrae*: predorsal 16 (2), 17 (5), precaudal+caudal 15+18 (1), 15+19 (5), 16+19 (1), within caudal peduncle 5 (4), 6 (3) in Cox’s Bazar specimens; predorsal 16 (1), 17 (5), precaudal+caudal 15+18 (1), 15+19 (4), 16+19 (1), within caudal peduncle 5 (4), 6 (2) in Rakhine specimens.

**Figure 2.**
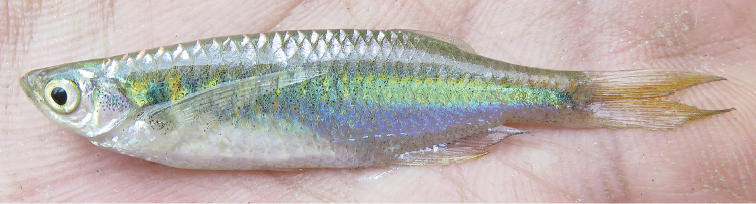
*Laubuka tenella*, paratype from the type locality, photographed alive immediately upon capture; specimen preserved, but not possible to match with particular preserved specimen.

Ground *colour* in formalin-fixed specimens (Figs 1A–B) pale yellowish white with diffuse grey or black markings except for round black cleithral spot size of pupil. Dorsum sparsely pigmented; brown stripe on dorsal midline from occiput to end of caudal peduncle. Thin black or brown stripe along middle of caudal peduncle or slightly longer, anteriorly replaced by 6–11 grey or brown short vertical bars, not reaching ventrally onto abdomen, less distinct in some larger specimens. A few black dots along middle of abdomen. Fins hyaline. Ethanol-fixed specimens (Fig. 1C), with silvery opercle and sides; vertical bars indistinct, dorsum grey, abdominal sides pale yellow; cleithral spot and lateral band black. Live specimens observed only in the type locality , with silvery reflections dorsally, abdomen white, sides blue, along middle a wide iridescent green cleithral spot flanked by golden (Fig. 2).

#### Etymology.

The specific name is a Latin adjective in diminutive form, *tenellus*, here in the meaning of delicate, referring to the small size and the soft, delicate consistency of fresh specimens.

#### Comparative morphometry.

**Figure 3.**
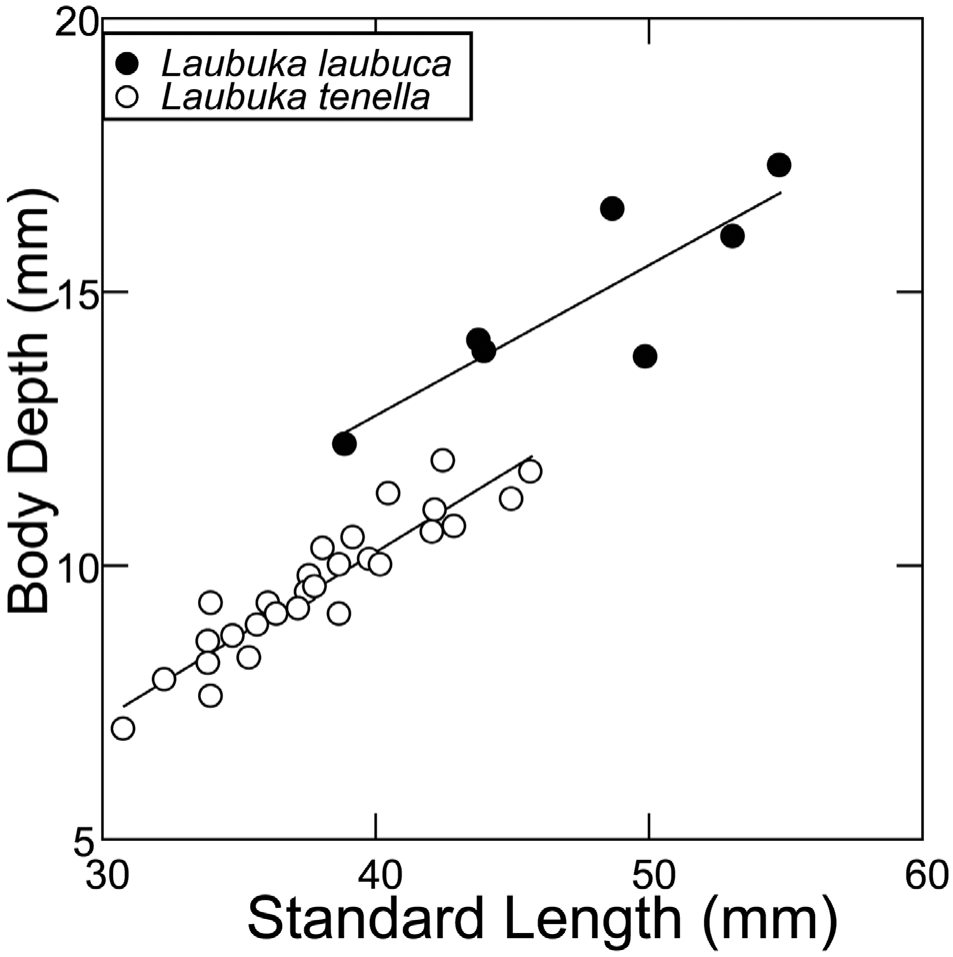
Body depth plotted against standard length in *Laubuka laubuca* and *L. tenella*.

*Laubuka tenella* is slightly more slender than *L. laubuca* of similar size (Tables 1–2, Fig. 3), but size differences and potential sexual dimorphism between the measurement series prevent a conclusive comparison. The sample of *Laubuka laubuca* (N=7) is too small to establish significant linear regression parameters.

**Table 1.** Morphometry of *Laubuka tenella*. Measurements are in per cent of standard length, except for standard length (in mm). SD, standard deviation; r, Pearson’s correlation coefficient; linear regression parameters calculated from measurements in mm, when ANOVA o= 0, and r> 0.9. HT = Holotype.

Measurements	N	HT	Min	Max	Mean	SD	r(SL)	slope (b)	intercept (a)
SL (mm)	29	42.1	30.8	45.7	37.8	3.8	–	–	–
Body depth	28	25.2	22.4	28.0	25.3	1.4	0.93	0.306	-1.989
Head length	28	20.9	20.9	23.9	22.9	0.7	0.94	0.194	1.335
Snout length	28	6.4	6.0	7.9	7.0	0.5	0.76	–	–
Head depth	28	14.7	13.6	15.8	14.7	0.6	0.91	0.128	0.698
Head width	28	11.9	11.8	13.3	12.6	0.4	0.95	0.100	0.968
Upper jaw length	28	7.1	7.1	8.6	7.8	0.4	0.86	0.061	0.607
Lower jaw length	28	10.7	9.8	13.0	11.2	0.8	0.74	0.069	1.616
Orbit diameter	28	7.6	7.2	9.1	8.3	0.5	0.81	–	–
Interorbital width	28	10.9	10.2	12.0	11.3	0.5	0.91	0.079	1.264
Caudal-peduncle length	28	15.4	12.6	16.2	14.1	0.9	0.80	–	–
Caudal-peduncle depth	28	9.0	8.2	10.8	9.3	0.6	0.89	0.110	-0.625
Dorsal-fin base length	28	14.3	11.3	14.4	12.7	0.8	0.83	–	–
Anal-fin base length	28	23.8	23.0	26.4	24.7	1.0	0.93	0.266	-0.721
Predorsal length	28	65.6	64.7	72.7	67.8	1.8	0.97	0.694	-0.619
Preanal length	28	61.8	61.7	69.1	64.6	1.6	0.98	0.701	-2.066
Prepelvic length	28	41.6	41.6	45.5	43.9	1.0	0.97	0.397	1.586
Pectoral-fin length	28	34.0	32.9	38.9	36.3	1.7	0.86	0.265	3.688
Pelvic fin length	28	16.4	14.9	19.1	17.1	0.9	0.87	0.159	0.439

**Table 2.** Morphometry of *Laubuka laubuca*. Measurements are in per cent of standard length, except for standard length (in mm). SD, standard deviation; r, Pearson’s correlation coefficient.

N	Min	Max	Mean	SD	r(SL)
8	38.9	55.6	48.6	5.9	
7	27.7	33.9	31.2	1.9	0.83
7	22.1	23.5	23.0	0.6	0.97
7	6.7	7.3	6.9	0.2	0.92
7	13.4	16.4	15.1	1.0	0.94
7	12.2	13.7	12.7	0.5	0.97
7	6.8	8.0	7.5	0.4	0.92
7	9.6	11.8	10.8	0.8	0.91
7	7.5	8.7	8.3	0.4	0.90
7	11.1	12.7	11.7	0.6	0.94
7	11.7	13.4	12.3	0.6	0.88
7	9.2	10.9	10.0	0.7	0.96
7	11.7	14.2	13.5	0.9	0.93
7	23.4	28.3	26.3	1.5	0.95
7	65.9	68.7	67.7	1.0	0.99
7	63.5	69.2	67.0	2.0	0.98
7	42.5	47.8	44.5	2.0	0.97
7	33.9	38.8	36.5	1.9	0.96
7	15.7	26.9	22.7	3.8	0.69

#### Phylogenetic characterization and relationships.

**Figure 4.**
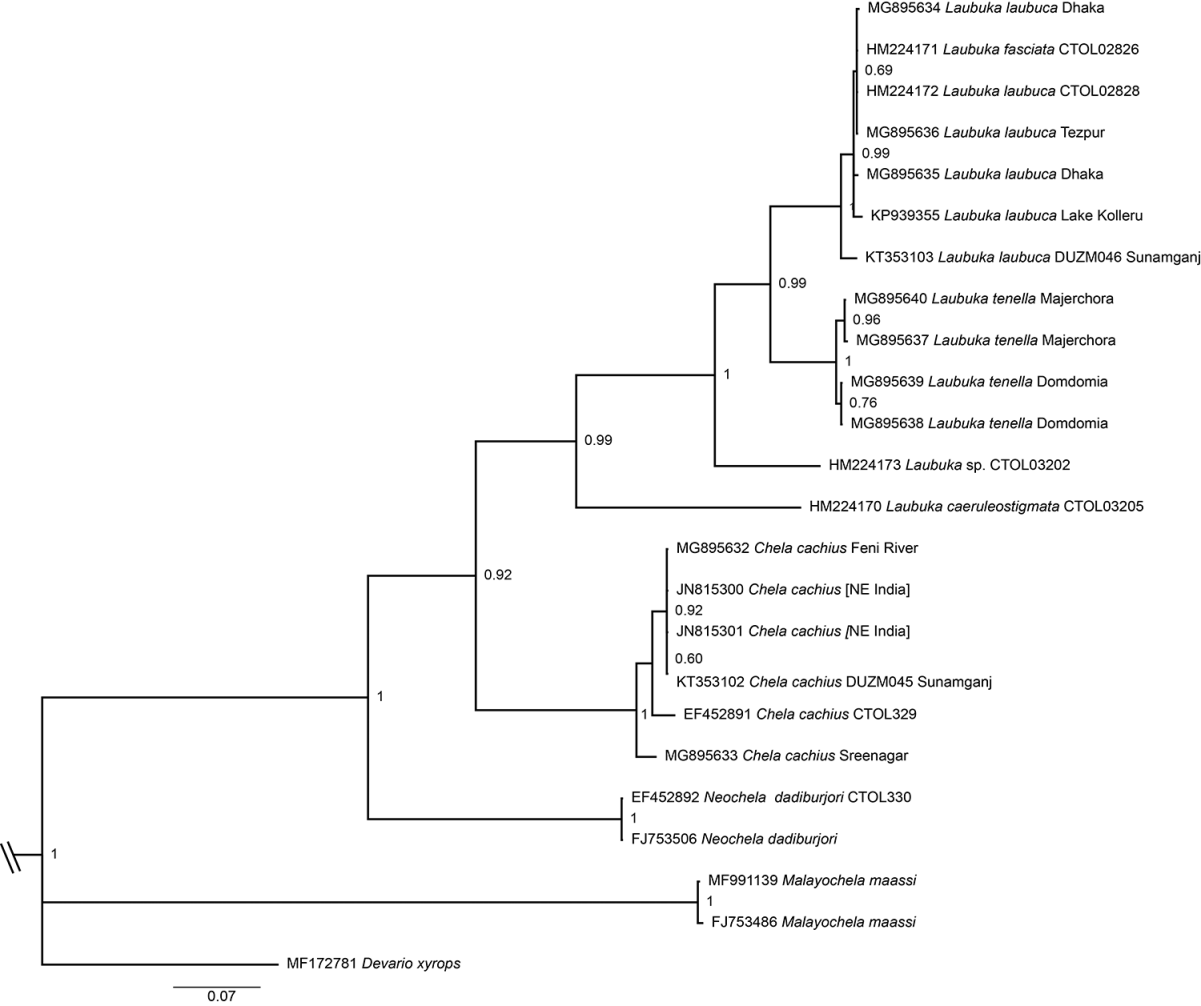
Phylogram of relationships of *Laubuka tenella* and similar taxa, based on a Bayesian analysis of the mitochondrial COI gene. Branch lengths are proportional to expected changes per site, visualizing estimated genetic distance. The scale bar represents number of expected substitutions per nucleotide site. Node labels show the Bayesian posterior probability of the clade. Terminal labels start with GenBank accession number and end with a locality indication when known. *Devario xyrops* and *Malayochela maassi* are outgroup taxa. HM224171, identified as *Laubuka fasciata* in GenBank, is apparently a misidentified *L. laubuca*. JN815300 and JN815301 with locality in the Bay of Bengal off Bangladesh and India, obviously in error. CTOL samples lack locality information.

The Bayesian phylogenetic analysis recovered *Laubuka tenella* as the sister species of *L. laubuca* (Fig. 4). COI sequences of *L. tenella* differed from the most similar sequences, in *L. laubuca*, by 9 % uncorrected p-distance. The within-species variation in *L. tenella* amounted to 0.9 %, between the Majerchora and Domdomia samples. Maximum pairwise *p*-distance in *L. laubuca* was 2.5% when the sequence KT353103 was included, and 0.9 % when excluded. Species delimitation methods confirmed reciprocal distinctness of *L. laubuca* and *L. tenella*: P ID(Liberal), the mean probability of making a correct identification of an unknown specimen of the focal species was reciprocally 0.97. At 6*10^-4^, Rosenberg’s P(AB) failed the null hypothesis that the combined clade (*L. laubuca*+*L. tenella*) represents a single species.

#### Geographical distribution and habitat.

**Figure 5.**
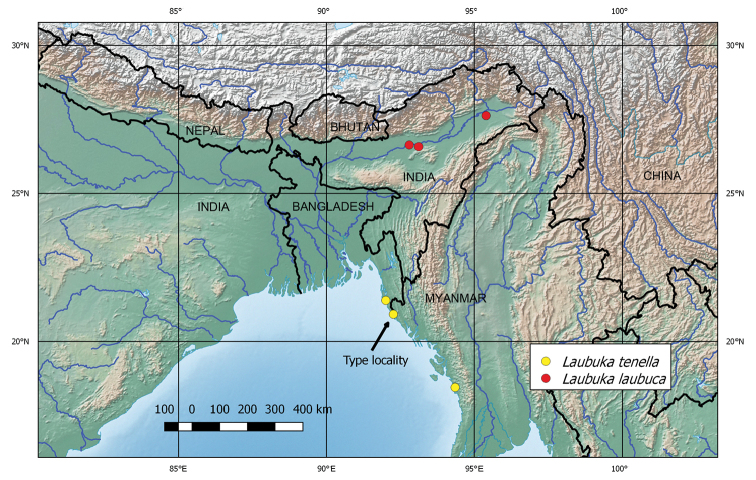
Map of collecting sites of *Laubuka laubuca* and *L. tenella*.

*Laubuka tenella* is known only from small streams in the vicinity of Cox’s Bazar and Teknaf in Bangladesh, and Thandwe in Myanmar (Fig. 5).

**Figure 6.**
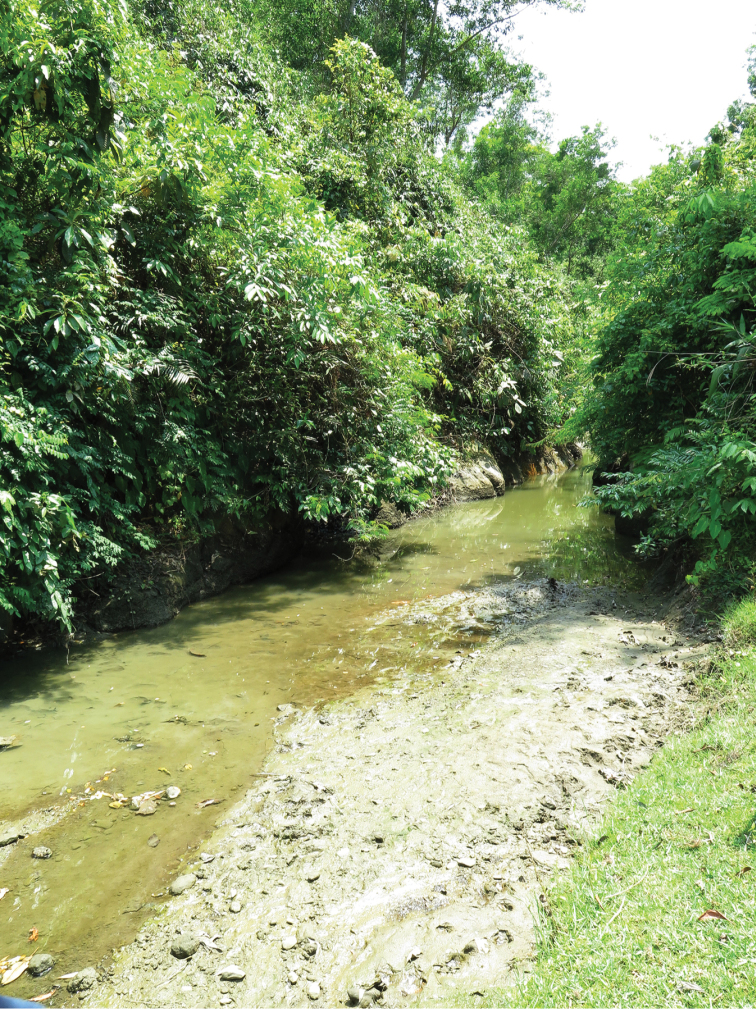
Domdomia stream, the type locality of *Laubuka tenella*, 9 May 2015.

Collections were made in the dry season when the streams had very little water. The type locality (Fig. 6) was in the lower course of the Domdomia stream, close to the mouth of the Naf River, flowing out of low forest into pasture associated with a village. At the time the stream was very shallow, not more than 10 m wide, with slightly turbid water and a bottom substrate of clay mixed with stones. The steep banks suggested that the water level in the monsoon season would reach a few meters higher. The associated fish fauna included *Aplocheilus panchax* (Hamilton) (Aplocheilidae), *Megalops cyprinoides* (Broussonet) (Megalopidae), *Acentrogobius caninus* (Valenciennes) (Gobiidae), *Eleotris melanosoma* Bleeker (Eleotrididae), *Dermogenys burmanica* Mukerji (Zenarchopterdae), and Oryzias cf. dancena (Hamilton) (Adrianichthyidae), reflecting proximity to the Naf River estuary. Other associated species in the Domdomia stream were identified as *Glossogobius giuris* (Hamilton) (Gobiidae), *Channa gachua* (Hamilton), *C. punctata* (Bloch) (Channidae), *Danio* sp., *Devario anomalus* Conway, Mayden & Tang, *Devario coxi* Kullander, Rahman, Norén & Mollah, *Esomus danrica* (Hamilton), *Pethia ticto* (Hamilton), *Puntius chola* (Hamilton), and *Rasbora rasbora* (Hamilton (Cyprinidae).

The Majerchora stream, in a hilly landscape with low forest near the sea, was very small, less than 1 m wide, and not more than about 30 cm deep. The water was only slightly turbid, flowing slowly over a bottom of sand and clay. Very few fish specimens were collected at this site; associated species were identified as *Danio* sp., *Devario coxi*, and *Pethia ticto* (Cyprinidae).

The locality in Myanmar was a stagnant pool in a desiccated small river, with leaf litter and sand on the bottom. The associated species were identified as *Anguilla* sp. (Anguillidae), *Aplocheilus panchax* (Aplocheilidae), *Channa* sp. (Channidae), *Danio aesculapii* Kullander & Fang, *Pethia* sp., *Puntius chola*, Rasbora cf. daniconius (Hamilton), *Rasbora rasbora* (Cyprinidae), and *Lepidocephalichthys berdmorei* (Blyth) (Cobitidae).

## Discussion

The first report of *Laubuka laubuca* from Bangladesh was by Rahman (1989), who illustrates it with a drawing copied from Day (1878: pl. 151, fig. 5), showing a specimen from Myanmar. The records by Rahman (2005) and Rahman and Chowdhury (2007), are illustrated with a photo that shows a specimen of *Esomus danrica* (Hamilton, 1822). No *Laubuka* were reported by Ahmed et al. (2013) from their survey of hillstream fishes in northern and southeastern Bangladesh. The distribution of *L. laubuca* within Bangladesh remains to be mapped. Based on the comparative material used here, *Laubuka laubuca* has a wide distribution in the Brahmaputra in Assam, whereas records from other areas need revision. *Laubuka brahmaputraensis* Kulabtong, Suksri & Nonpayom, 2012, is the only other species of *Laubuka* reported from Bangladesh. It was described on the basis of aquarium specimens without precise collecting locality, but said to be from the Brahmaputra basin in Bangladesh (Kulabtong et al. 2012), and has been considered to be a possible junior synonym of *L. laubuca* (Kottelat 2013). *Laubuka tenella* is distinct from *L. laubuca* in colour pattern and mitochondrial DNA, and there is no indication that it has been misidentified as *L. laubuca* in earlier literature.

The Cox’s Bazar and Rakhine samples of *L. tenella* share the same colour pattern and proportional measurements, but differ slightly in frequencies of anal-fin ray counts. Ranges overlap, however, and the anal fin count shows intraspecific variability also in other species of *Laubuka*. There is no variation in dorsal-fin count or circumpeduncular scale count, but these counts (ii.8½ and 12, respectively) are shared with most other species in the genus. The miniature species *Laubuka fasciata* ii.7½ dorsal-fin rays, and 10 circumpeduncular scales. Pethiyagoda et al. (2008) reported dorsal-fin rays ii.9½ in some Sri Lankan species and we observed this count in a specimen of an unidentified species of *Laubuka* from Kerala (NRM 12218) .

*Laubuka khujairokensis*, *L. latens*, *L. laubuca*, and *L. siamensis* are characterized by absence of short dark bars anteriorly on the side. However, Das (1939: fig. 1) figured a specimen identified as *L. laubuca* from the Damodar River near Hazaribagh (Hugli River drainage), which shows short indistinct vertical bars on the side, but no lateral stripe. Otherwise, a series of short vertical bars on the side as in *L. tenella*, have been reported only from Sri Lankan *Laubuka*, by Pethiyagoda et al. (2008). *Laubuka trevori* has a narrow dark stripe along the side, stated to be less distinct anteriorly (Knight 2015), but apparently no vertical bars. Consequently, *L. tenella* is diagnosed on the basis of colour pattern and, as verified from Bangladeshi material only, the COI sequence. *Laubuka tenella* is also relatively slender, and may differ in that regard from *L. laubuca* (Fig. 3, Tables 1–2) , and Sri Lankan species (body depth 32.8–34.6 % SL in *L. ruhuna*, 27.9–32.4 % in *L. varuna*, 27.2–30.3 % in *L. lankensis* according to data in Pethiyagoda et al. (2008).

*Laubuka brahmaputraensis* was described from three specimens collected by an aquarium fish collector and said to be from Brahmaputra but without precise locality. The published photograph (Kulabtong et al. 2012; fig. 1) of the holotype, preserved in 1995, suggests that it is in a poor state of preservation, similar to specimens kept long time in buffered formalin. The photo shows a trace of the cleithral spot but no other markings. The diagnosis and description do not include characters separating from *L. laubuca* reported here from Bangladesh and northeastern India. Kulabtong et al. (2012) did not have specimens of *L. laubuca* for comparison, but based their concept of *L. laubuca* on the meristic data in “Rahman (2003)” which is apparently an error for Rahman (2005). The validity of this comparison is doubtful.

Rahman’s (2005) description is not in accord with *Laubuka* from Bangladesh or northeastern India, but also not compatible with *Esomus danrica* (Hamilton, 1822), the species on the illustration of *L. laubuca* in Rahman (2005). Rahman’s fin counts are compatible with most species of *Laubuka*, but the counts of 34–36 lateral-line scales, and 20–21 predorsal scales stand out. The description is almost the same as in Rahman (1989) and the counts are identical. The high lateral-line counts and predorsal scale counts seem to be based partly or entirely on earlier literature. Silas (1958) identified *L. laubuca* from a wide geographical area, including India, Sri Lanka, Myanmar and adjacent south-east Asia, resulting in a lateral line count range of 31–37, as cited by Talwar and Jhingran (1991). Silas (1958) did not report specimens from Bangladesh, and his specimens from Myanmar were from east of the Rakhine Yoma. Jayaram (1981, 1999) stated the lateral line count to be “34 to 37, large, not many”, a count that may go back to the count of 34–37 in Day (1878: 598). Day’s figure, pl. 151, fig. 5, is stated to show a specimen from Burma and most likely does not show *L. laubuca*. Day’s description also covers material from India and Sri Lanka. Day’s Myanmar specimens, included in *L. laubuca* by Silas (1958), now in the BMNH, are from Mawlamyine, Sittaung River, and Mandalay (Silas 1958). Day’s image was copied by later authors to illustrate *L. laubuca*, e.g., in Jayaram (1981) and Rahman 1989). Pethiyagoda et al. (2008) reported elevated counts of 34–37 lateral line scales in *L. lankensis* and 34–36 in *L. insularis*, but only and 31–33 in *L. varuna* and *L. ruhuna*.

**Figure 7.**
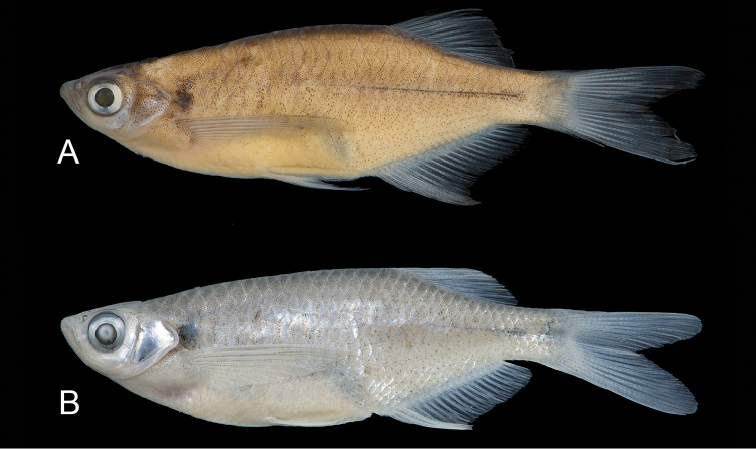
*Laubuka laubuca*. **A** NRM 57234, 53.1 mm SL. India: Brahmaputra River drainage: vicinity of Dibrugarh **B** NRM 67317 mm SL, preserved in 95% ethanol. Bangladesh: vicinity of Dhaka.

Because earlier concepts of *L. laubuca* were highly inclusive, including several species, and also including information copied from literature sources, published information on *L. laubuca* cannot be relied on for diagnosis of the species. Based on the locality information provided by Hamilton (1822; Day, 1877), it seems reasonable that the species of *Laubuka* present in West Bengal and Assam represents *L. laubuca* in the strict sense. This species, with 30–33 lateral-line scales has a colour pattern that includes a distinct vertically oriented cleithral spot, and a thin black stripe from the middle of the side which is extended caudad but not reaching to the caudal-fin base, and which is slightly expanded on the middle of the caudal peduncle. This colour pattern is illustrated by Pethiyagoda et al. (2008: fig. 13), and Lalramliana et al. (2017: fig. 2), and in Fig. 7A–B). Except for the absence of data in Kulabtong et al. (2012), *L. brahmaputraensis* agrees with *L. laubuca* in this strict sense, suggesting that *L. brahmaputraensis* is a junior synonym of *L. laubuca*.

*Danio menoni* Barman, 1985 from Andhra Pradesh (Barman 1985) was identified by Tilak and Jain (1987) as a junior synonym of *L. laubuca*. The image in Barman (1985) shows clearly that it is a species of *Laubuka*, but it has a distinct triangular caudal spot similar to a specimen from Kerala in our comparative material, and may represent a valid species of *Laubuka*.

*Perilampus perseus* M’Clelland [1839: 395, pl. 46, fig. 5 (erroneously stated to be pl. 48, fig. 5 in the text]; as *P. persus* in the index)] was listed as a questionable synonym of *L. laubuca* by Day (1878), and has not been recognized as a distinct species since. The distribution was given as “Assam, and probably Bengal”, and the description of the only specimen said to be “so much injured that I am unable to determine with accuracy the number of scales and caudal fin rays” (M’Clelland, 1839). Knight (2016) considered M’Clelland’s illustration to be reminiscent of *Salmostoma*, but particularly the long pelvic fin rather supports the identification as a species of *Laubuka*. Based on the locality information by M’Clelland (1839), *P. perseus* is very likely to be a junior synonym of *L. laubuca. Perilampus guttatus* M’Clelland, 1839, is an unneeded replacement name for *Cyprinus laubuca* Hamilton, 1822 (= *L. laubuca*).

Among the Sri Lankan species of *Laubuka* described by Pethiyagoda et al. (2008), *L. lankensis*, and *L. insularis* were distinguished from *L. ruhuna* and *L. varuna* by the presence of tubercles on the lower jaw. Those structures are, however, neuromast fields, as observed in *L. insularis* and other species of *Laubuka*, and should be expected from *L. varuna* and *L. ruhuna* as well. Specimens identified as possibly *L. varuna* (LZM 962/5438) have well developed lower jaw neuromast fields. Lower jaw neuromast sensory fields may be a unique character of *Laubuka*, but have not yet been studied at organ level. The diagnoses of the Sri Lankan species, based mainly on scale counts, fin lengths and body depth, make it difficult to separate them from continental Asian congenerics, but it seems unlikely that any of them is conspecific with species in the northern part of India and Bangladesh.

Günther (1868: 331) described *Eustira ceylonensis* as a new genus and species based on six specimens from Ceylon, purchased of Mr Cuming [Hugh Cuming, 1781–1865]. Day (1878: 599) included *E. ceylonensis* in *Perilampus*. Day’s *Perilampus* equates *Laubuka*+*Chela*. His description of *P. ceylonensis* is based on data from Günther (1868); apparently Day had not examined Günther’s specimens. Silas (1957) synonymized *E. ceylonensis* with *Danio malabaricus* (Jerdon, 1849) (now *Devario malabaricus*) based on his own examination of the presumed type series. Silas also provided a line drawing, supplied by Ethelwynn Trewavas, of a specimen stated to be holotype of *E. ceylonensis*, and which seems to show a specimen of *Devario*. It is noteworthy that the data from the specimens examined by Silas disagree with Günther’s description, while in the same volume Günther (1868: 282–283) lists specimens of *D. micronema* (Bleeker, 1863) from Ceylon (“*a, b, c, d-e, f-h*. Adult and half-grown”), of which *a* is stated to have been purchased of Mr. Cuming; and also material of other species of *Danio*/*Devario*. Silas’s assumption that Günther was unable to distinguish between *Laubuka* and *Danio*/*Devario* may have been somewhat precipitant. It seems rather that a sample of *D. micronema* (as identified by Günther) was mistaken by Silas and Trewavas for the type series of *E. ceylonensis*.

Günther’s description of *E. ceylonensis* is compatible with characters of *Laubuka*. Günther’s diagnosis of the genus, referring to “entire abdominal edge being trenchant”, i.e. keeled, as in *Laubuka*, stands in contrast to the rounded abdomen in *Devario*. The characters “pectorals elongate” (characteristic of *Laubuka*; not particularly long in *Devario*); “barbels none” (as in *Laubuka*; usually both rostral and maxillary barbels present in *Devario*), and “ventrals well developed” (long in *Laubuka*, not particularly so in *Devario*) also suggest a different genus than *Devario*. Günther’s description of the species *E. ceylonensis* excludes *Devario* by reference to absence of a symphyseal knob of the lower jaw, which is prominent in *Devario* and *Chela*, and silvery colour, not recorded from any Sri Lankan *Devario*. The species description, however, contains a statement that is difficult to reconcile with *Laubuka* or *Devario*: “Pectoral fin shorter than the head, extending to the ventral.” This statement also seems to be at odds with the diagnosis of *Eustira*, which suggests a long pectoral fin, even if “elongate” could also be understood as slender. Other parts of the description of *E. ceylonensis* are compatible with both *Laubuka* and *Devario*, as well as other cyprinid genera. The anal-fin count of 17 is low for *Laubuka*, which usually have about 20 anal-fin rays, but it may exclude the first two unbranched rays, which are difficult to discern without manipulation.

The jar with the syntypes of *Eustira ceylonensis* could not be located in the BMNH collection (J. Maclaine, R. Britz, pers. comm.). The specimens incorrectly considered as the type series of *Eustira ceylonensis* are catalogued as BMNH 1852.2.19.130–132, 1853.12.24.6, 1864.4–11.33, and are identified by us as tentatively representing *Devario malabaricus* sensu Pethiyagoda (1991). *Eustira ceylonensis* is probably a senior synonym of one of the species of *Laubuka* reported by Pethiyagoda et al. (2008) from Sri Lanka, but Günther’s description does not contain information that can be used to identify which of those species is affected.

Although the Myanmar and Bangladeshi samples of *Laubuka tenella* could not be separated by morphological characters, it may be noted that meristics and proportional measurements are very conservative in *Laubuka*. The colour pattern is unique to *L. tenella* but similar to that of other species. A longitudinal dark stripe occurs in several species, although usually narrower than in *L. tenella*, and short vertical bars anteriorly on the side is illustrated for *L. insularis* and *L. varuna* in Pethiyagoda et al. (2018). The combination of a posterior dark stripe and anterior series of short vertical bars is unique for *L. tenella*. The colour pattern is definitely different from that of *L. fasciata* and *L. parafasciata* in which there is a horizontal dark stripe along the middle of the side (Knight 2015: fig. 15; Lalramliana et al. 2017: fig. 2). Specimens referable to *L. laubuca* have plain sides except for a distinct dark cleithral spot, a very narrow dark stripe posteriorly on the caudal peduncle and, in juveniles only, a dark spot at the base of the caudal fin. In *L. siamensis* there are indistinct vertical bars anteriorly on the side, and posteriorly on the side a dark stripe ending in a triangular blotch on the caudal-fin base.

**Figure 8.**
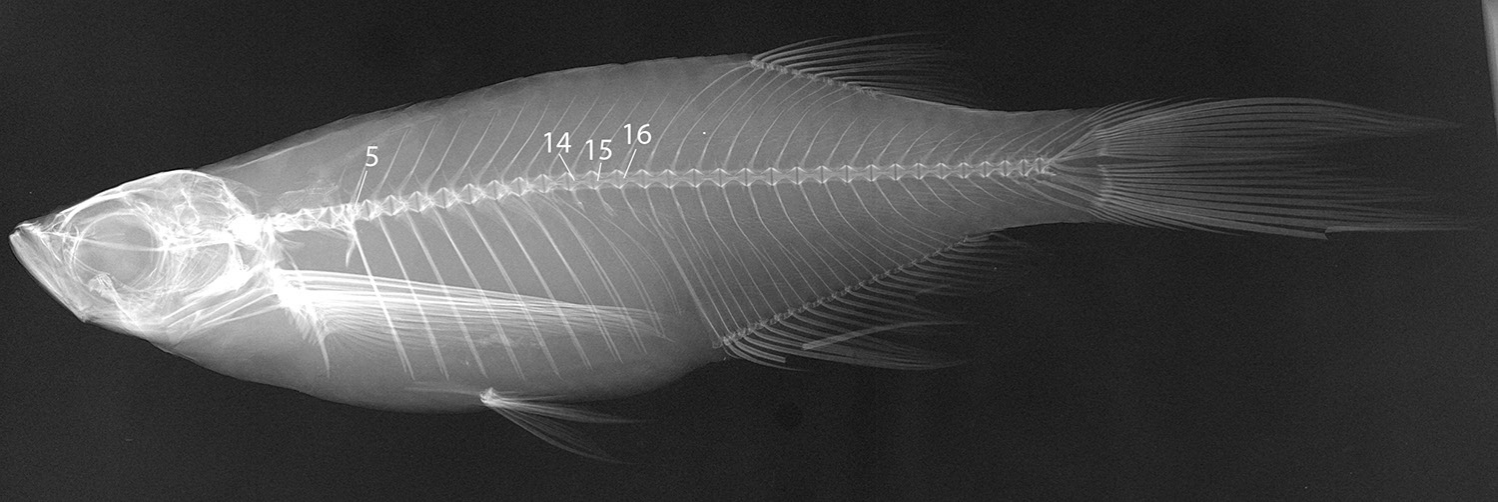
X-radiograph of *Laubuka tenella*. Significant vertebrae indicated with sequential number. 5 = Fifth precaudal vertebra (= 5^th^ abdominal/trunk vertebra); 14 = 14^th^ precaudal vertebra, anterior of two precaudal vertebrae with haemapophyses and non-articulating ribs/ribs absent (intermediate vertebrae of Naseka 1996); 15 = last precaudal vertebra; 16 = first vertebra with haemal spine, which is first caudal vertebra of Günther (1880), Naseka (1996), Hubbs and Lagler (2007), and Bird and Mabee (2003), and the last preanal vertebra of Naseka 1996).

Lalramliana et al. (2017) considered 16 precaudal vertebrae to be diagnostic for *Laubuka parafasciata*, contrasting with 14 in other *Laubuka*. The count of 14 was probably based on Pethiyagoda et al. (2008) who reported 14 precaudal vertebrae in *Laubuka* as diagnostic from *Chela cachius* with 17. Our specimens of *Laubuka* possess 15 or 16 vertebrae anterior to that with the first distinct haemal spine (Table 3). Typically, vertebrae 5–14 bear ribs, vertebrae 15 is a short transitional vertebra without ribs or distinct haemal spine, vertebra 16 presents a haemal spine, and vertebra 17 has a haemal spine inserted posterior to the ascending first anal-fin pterygiophore (Fig. 8) There are no major differences in vertebral counts between species of *Laubuka*, as also suggested by preliminary data from a few species (Table 3).

**Table 3.** Vertebral counts in *Laubuka tenella* and comparative material of *Laubuka*.

Species	N	14+19=33	15+18=33	15+19=34	15+20=35	16+18=34	16+19=35
*L. tenella*	13		2	9			2
*L. parafasciata*	6			4	1	1	
*L. laubuca*	2	1		1			
*L. insularis*	2				2		
*L. siamensis*	5			1	4		

The terminology of vertebral elements in teleosts is diverse, with confusing synonymy. Günther (1880, 1886) and Hubbs and Lagler (2007; and editions from 1947 onwards) provided the most influential list of definitions of measurements and counts in fish taxonomy. Günther distinguished between abdominal and caudal vertebrae, and Hubbs and Lagler between precaudal and caudal vertebrae, both publications considering the first caudal vertebra to be that bearing a definite haemal spine. Günther (1880) stated more precisely: “the caudal vertebrae differ from the abdominal in having the haemapophyseal elements converted into spines similar to the neurals”.

Naseka (1996) proposed a vertebral column formula recognizing the main divisions of abdominal and caudal vertebrae. Naseka’s abdominal vertebrae include intermediate vertebrae which are vertebrae with parapophyses but no articulating ribs. His caudal vertebrae include preanal vertebrae with a haemapophysis anterior to the first (or first interhaemal) anal-fin pterygiophore, and postanal vertebrae, which are those posterior to the first anal-fin pterygiophore. Bird and Mabee (2003) mapped vertebral elements in *Danio rerio*, distinguishing between Weberian (within the Weberian complex), precaudal (from vertebra 5 to the one before that bearing the first haemal spine, “…vertebrae composed of centra, neural arches and spines, parapophyses, and ribs”), caudal (from that with the first haemal spine to the one before the caudal-fin vertebrae, “…vertebrae […] composed of centra, neural arches and hemal spines”), and caudal-fin (those supporting the caudal-fin rays) vertebrae.

In cypriniforms, there are always four vertebral centra within the Weberian apparatus; posterior vertebrae may vary in number and morphology. In *Laubuka* the Weberian vertebrae are followed by trunk vertebrae with long pleural ribs and usually three vertebrae with no ribs attached. Of these latter three, the anterior two have parapophyses, a haemal arch, and non-articulating free pleural ribs, whereas the third is slender and extends ventrad as a haemal spine immediately anterior to the long first anal-fin pterygiophore or haemapophysis (Fig. 8). On X-radiographs of fish specimens, the shape of the posterior trunk vertebrae commonly cannot be decided, but, as in *Laubuka*, their identity is deduced from the relative position, presence or absence of attached ribs, and the relative length of the haemapophysis/haemal spine. Roberts (1989), working with X-radiographs, suggested to count as caudal vertebrae those posterior to the first anal pterygiophore. This method was followed by e.g., Kullander and Britz (2002), and probably also Lalramliana et al. (2017).

Differences between authors in reporting vertebral counts can be attributed to different interpretations or definitions of the vertebrae in the transitional zone between trunk and tail vertebrae. The precaudal count in Pethiyagoda et al. (2008) apparently refers to the Weberian plus rib-bearing vertebrae, whereas in Lalramliana et al. (2017) it apparently refers to the Weberian plus remaining trunk vertebrae including the vertebra with the first haemal spine, i.e., all preanal vertebrae (but not abdominal or precaudal vertebrae as in Günther (1880) or Hubbs and Lagler (2007).

Vertebral numbers and the delimitation of trunk and tail vertebrae are useful taxonomic markers and may explain body proportions. Limitations in resolution of X-ray images or difficulties to determine the first haemal spine may influence the classification of trunk and tail vertebrae, however. It seems relevant in danionin cyprinids to follow Bird and Mabee (2003) in distinguishing between precaudal vertebrae (i.e. the ones anterior to the vertebra immediately anterior to the first anal-fin pterygiophore, understood as bearing the first haemal spine, but in contrast to Bird and Mabee including the four Weberian vertebrae), and caudal vertebrae (all vertebrae posterior to and including this vertebra, including caudal-fin supporting vertebrae, i.e., the the last half-centrum= compound centrum or preural+ural1) (Fig. 8). This division agrees with that of Günther (1880, 1886) and Hubbs and Lagler (2007). In groups in which the shape of transitional trunk vertebrae (Naseka’s intermediate vertebrae) is difficult to classify, some other method to count or to distinguish the vertebrae may be more practical. Because the subdivisions are based on convention only, any other method than those described above are equally valid, but the particular method of counting vertebrae needs to be explained for every instance to ensure that counts are consistent and comparisons achievable.

In the phylogenetic analysis, *Laubuka tenella* came out as sister group to *L. laubuca*, which has a complementary geographical distribution west of *L. tenella*. Only few sequences of *Laubuka* were available from GenBank to supplement our material from India and Bangladesh, and among them several lacked locality information (Fig. 4). Incorporation of additional taxa, e.g., from Sri Lanka, Myanmar and Indochina may alter significantly the pattern shown in Fig. 4. Nevertheless, the position in the tree (Fig. 4) and the 9 % *p*-distance difference from the most similar species, are strong indicators of species distinctness of *L. tenella*. One of the downloaded GenBank sequences, KT353103, from northern Bangladesh, and identified as *L. laubuca* in GenBank, is resolved in the *L. laubuca* clade (Fig. 4), but as sister to the remaining specimens. A possible explanation is that it represents one more species of *Laubuka* in the region, but could also reflect wide genetic variation in *L. laubuca*.

*Laubuka tenella* is known so far only from three localities, and DNA data are available from only two localities, representing two distinct haplotypes. It seems plausible to consider the localities to be part of a continuous distribution, but the differences in the COI sequence between the two Bangladeshi localities may be an indication of fragmentation. The coasts of extreme southeastern Bangladesh and southwestern Myanmar are ichthyologically underexplored. The Rakhine Yoma is a hill range separating the fish fauna in the lowlands to the west from that of the Irrawaddy basin, exemplified by several endemic species from western Rakhine Yoma (e.g., Kullander 2015, Kullander and Britz 2015). This lower costal region extends into southeastern Bangladesh, and it is not surprising to find the same species both in southwestern Myanmar and southeast, although *L. tenella* may be the first species reported as endemic for this particular region. *Devario anomalus*, in the Cox’s Bazar district, and *D. xyrops* Fang & Kullander, 2009, on the Western slope of the Rakhine Yoma, are two very similar sister taxa, which are considered as distinct species (Kullander et al. 2017), but with a combined distribution similar to that of *D. tenella*.

## Supplementary Material

XML Treatment for Laubuka tenella
